# Evaluation of Ternary Mobile Phases for the Analysis of Carbonyl Compound Derivatives Using High-Performance Liquid Chromatography

**DOI:** 10.1100/tsw.2011.4

**Published:** 2011-01-05

**Authors:** Duy Xuan Ho, Ki-Hyun Kim

**Affiliations:** Department of Environment and Energy, Sejong University, Seoul, Korea

**Keywords:** high performance liquid chromatography (HPLC), carbonyl compounds (CCs), mobile phase, CCs-DNPH(o) derivatives, ternary mobile phase

## Abstract

In this study, the feasibility of ternary mobile phases was examined in a high-performance liquid chromatography (HPLC)-based analysis of carbonyl compounds (CCs). To test the performance of different ternary phases, the liquid phase standards containing a 15 aldehyde/ketone-DNPH(o) mix were analyzed through a series of five-point calibration experiments. For this comparison, three types of ternary mobile phases were prepared initially by mixing water (W) with two of the following three organic solvents: isopropanol (I), methanol (M), and tetrahydrofuran (T). The resulting three types of ternary phases (named as WIM, WTM, and WIT) were tested and evaluated in relation to the water content or in terms of methanol-to-water ratio (M/W). The results derived by the three ternary phases revealed that the optimal resolution was attained near maximum water content, while those of WIT consistently suffered from poor resolution problems. The relative performances of WIM and WTM phases, if assessed by three key operating parameters (sensitivity, retention time, and resolution), were found to be reliable for most selected CCs with the decreasing M/W ratio.

## INTRODUCTION

Carbonyl compounds (CCs), including low-molecular-weight aldehydes and ketones, are ubiquitously found in diverse environmental settings. As CCs can exist in a heterogeneous mixture of gas, liquid, and particulate phases, their pollution is observed in all types of environmental compartments, i.e., the atmosphere, oceans, and soil systems[1]. These compounds are also known to initiate the formation of photochemical smog or to sustain reactions leading to ozone formation. In addition, they act as the precursors of free radicals, ozone, peroxyalkyl nitrates, peroxyalkylbenzylates, and organic acids[2], or as the products of photo-oxidation of gas-phase hydrocarbons[3]. Furthermore, CCs are one of the major hazardous pollutants in the urban atmosphere due to their potential impact on human health, including toxins, corrosives, irritants, mutagens, and carcinogens (e.g., formaldehyde and acetaldehyde)[1,4]. As a result, CCs have commonly been investigated in numerous interdisciplinary studies covering environmental pollution, health-related studies, food chemistry, and industrial safety[5].

The most abundant CCs in ambient air are usually represented by formaldehyde and/or acetaldehyde[6,7,8]. As such, changes in their concentration levels are often reflected by local photochemical activities and the consequential air pollution[9]. Due to the important role of CCs in air quality maintenance, efforts to reduce their concentration levels have drawn a great deal of attention as one of the most imminent issues calling for intervention in many countries[10]. Possible sources of CCs are diverse enough to include natural vegetation, wood combustion, ethanol-fueled vehicles, industrial plants, cigarette smoke, incomplete combustion of biomass, fossil fuel burning, candles, and incense[9,11,12]. Carbonyls can also be formed by atmospheric oxidation of gaseous polycyclic aromatic hydrocarbons (PAHs) or volatile organic compounds (VOCs) in liquid or particulate phases[1]. Indoor sources, such as insulation, tobacco smoke, furniture, and particle board, can also contribute to its budget. Because of unique relationships between CCs, their concentration ratios (e.g., formaldehyde to acetaldehyde and/or acetaldehyde to propionaldehyde) are often evaluated to explain the processes affecting their relative distributions[13,14]. As such, the monitoring of CCs in the atmosphere is also the primary way to explore the oxidation pathway of VOCs[13,14].

Despite the recognition of the potential role of CCs in atmospheric chemistry, there have been many limitations in the identification of unknown carbonyl species and their quantification at trace-level quantities[15]. Many researchers have developed techniques to determine CCs and volatile organics in air through the assessment of their behavior and exposure pathways over a variety of spatial and temporal scales[16,17,18]. One of the most common trends in their analysis can be summarized as the use of a hydrazine reagent. Such applications employed a substrate coated with various reagents (e.g., sodium hydrogen bisulfite, 2,4-dinitrophenylhydrazine, chromotropic acid, or N-methyl-4-hydrazino-7-nitobenzofurazan) to chemically trap the analytes[19,20,21,22,23,24]. Their instrumental detection is then commonly completed by high performance liquid chromatography (HPLC) with ultraviolet-visible spectroscopy (UV-VIS) detectors or gas chromatography (GC) methods[12]. However, the use of the GC method is known to be affected by such factors as the decomposition of CC derivatives or the lack of proper adsorptive materials (e.g., [12]). As the GC technique has not gained full confidence, its application has been confined to a number of well-defined CCs. Although photometric or fluorescence spectroscopy can also be employed for the analysis of CCs (e.g., formaldehyde and acetone) in air samples, these techniques can suffer from low sensitivity or interfering components (e.g., ozone)[5].

As a means to overcome the various technical limitations in CC analysis, the HPLC method has been validated most intensively via *in situ* derivatization of carbonyls with 2,4-dinitrophenylhydrazine (DNPH[i])[25]. In an effort to find the most appropriate elution conditions for CCs, HPLC-based methods have been developed through the diversification of mobile phases with various compositions. The supplementary laboratory efforts to develop the HPLC method have, in fact, greatly contributed to the accumulation of a database for atmospheric CCs. In order to help describe the optimum operation conditions of the HPLC system, we undertook a series of experiments in which the relative performance of its mobile phases was tested[26]. As the main goal of this study, we provide and discuss experimental results derived using these ternary mobile phases with the diverse composition for the analysis of CCs.

## MATERIALS AND METHODS

### Solutions and Preparation of the Working Standard (WS)

In this article, HPLC-based analytical methods were investigated using standards containing a liquid phase mixture of 15 aldehyde/ketone-DNPH. As shown in Table 1, these 15 CCs consist of formaldehyde (FA), acetaldehyde (AA), acrolein (AR), acetone (AT), propionaldehyde (PA), crotonaldehyde (CA), butyraldehyde (BA), benzaldehyde (BZ), isovaleraldehyde (IV), valeraldehyde (VA), o-tolualdehyde (OT),

**TABLE 1**
**List of 15 Target CC Derivatives Investigated in this Study**

| Order[a] | Name of Target Compounds | | Molecular Formula | MW[b] | Density | $t_R$[c] | Structure |
|---|---|---|---|---|---|---|---|
| | Full Name | Short Name | | | | | |
| 1 | Formaldehyde | FA | HCHO | 30.03 | 1.083 | 3.2 |  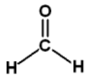 |
| 2 | Acetaldehyde | AA | $CH_3CHO$ | 44.05 | 0.788 | 3.8 |  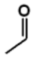 |
| 3 | Acrolein | AR | $CH_2CHCHO$ | 56.06 | 0.839 | 4.4 |  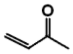 |
| 4 | Acetone | AT | $CH_3COCH_3$ | 58.08 | 0.790 | 4.6 |  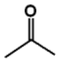 |
| 5 | Propionaldehyde | PA | $CH_3CH_2CHO$ | 58.08 | 0.798 | 4.8 |  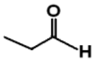 |
| 6 | Crotonaldehyde | CA | $CH_3CHCHCHO$ | 70.09 | 0.858 | 5.6 |  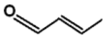 |
| 7 | Butyraldehyde | BT | $CH_3CH_2HCH_2CHO$ | 72.11 | 0.817 | 6.2 |  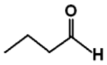 |
| 8 | Benzaldehyde | BZ | $C_6H_5CHO$ | 106.12 | 1.042 | 6.9 |  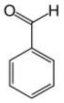 |
| 9 | Isovaleraldehyde | IV | $(CH_3)_2CHCH_2CHO$ | 86.13 | 0.821 | 7.9 |  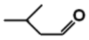 |
| 10 | Valeraldehyde | VA | $CH_3(CH_2)_3CHO$ | 86.13 | 0.810 | 8.3 |  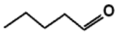 |
| 11 | o-Tolualdehyde | OT | $CH_3C_6H_4CHO$ | 120.15 | 1.039 | 8.7 |  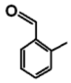 |
| 12 | m-Tolualdehyde | MT | $CH_3C_6H_4CHO$ | 120.15 | 1.019 | 8.9 |  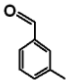 |
| 13 | p-Tolualdehyde | PT | $CH_3C_6H_4CHO$ | 120.15 | 1.015 | 9.1 |  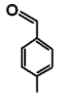 |
| 14 | Hexaldehyde | HA | $CH_3(CH_2)_4CHO$ | 100.16 | 0.814 | 11.5 |  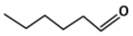 |
| 15 | 2,5-Dimethylbenzaldehyde | DB | $(CH_3)_2C_6H_3CHO$ | 134.18 | 0.950 | 11.7 |  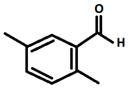 |

[a] Carbonyl number: derived based on retention time ($t_R$).

[b] Molecular weight.

[c] Retention time based on ACN:water (70:30) mobile phase.

m-tolualdehyde (MT), p-tolualdehyde (PT), hexaldehyde (HA), and 2,5-dimethylbenzaldehyde (DB). In the course of this study, a method based on the formation of a derivative mixture was consistently employed for the analysis of carbonyls with the support of the UV-VIS detector. For the purpose of this study, a number of ternary mobile phases were prepared and tested against the HPLC systems to evaluate the optimum separation conditions of the selected CCs. Initially, preliminary experiments were conducted to study binary, ternary, and quaternary mobile phases (e.g., [26]). In this study, we extended our efforts to focus on various combinations of ternary phases. The results of the preliminary experiments confirmed that the tests for quaternary mobile phases demand a high throughput size, while those of binary phases have limited selectivity. In light of such complexity involved in the selection of the mobile phase types, we attempted to derive the optimized operation conditions of HPLC with these ternary mobile phases. To this end, all the solvents were prepared to form three types of ternary mobile phases by mixing water (W) with two of the following three organic solvents: methanol (MeOH), tetrahydrofuran (THF), and isopropanol (IP). Hence, three types of ternary phases can be derived with the combination of (W): (1) WIM by mixing with isopropanol (I) and methanol (M), (2) WTM for tetrahydrofuran (T) and methanol (M), and (3) WIT for isopropanol (I) and tetrahydrofuran (T). The results of all ternary phase experiments were put together and then evaluated to derive the best qualification of CCs, e.g., in terms of resolution, sensitivity, and run time. To separate the sum of 15 CCs by our HPLC system, the WS were eluted through a Hichrome C18 column (250 mm × 4.6 mm × 5 μm) at a constant flow rate of 1.5 mL $min^{-1}$.

Our experiments were designed to explore the three types of ternary mobile phases for the optimum detection of 2,4-dinitrophenylhydrazone (DNPH[o]) derived from the pure 2,4-dinitrophenylhydrazine (DNPH[i]). (Here, the lower-case letters of i and o denote before [-zine] and after the derivatization [-zone], respectively.) The performance of these ternary mobile phases was examined in reference to the most commonly used mobile phase combination, known as the binary mixture of water and acetonitrile (ACN) (e.g., water:ACN [30:70]). In order to prevent problems with contaminants or bio-organisms on manifestation of results, ultrapurified water was supplied via a water purification system. ACN and MeOH were supplied by J.T.Baker (USA), while THF and IP were purchased from Dae-Jung Chemicals & Materials (Korea).

The primary standard (PS) of CCs was purchased as a mixture of 15 CCs DNPH(o) with T011/IP-6A aldehyde/ketone-DNPH(o) mix (catalog number 47285-U, Supelco). The concentration of all CCs in this PS was preadjusted to 15 μg $mL^{-1}$. In addition, the PS for each individual compound was also purchased from Aldrich-Sigma, with the purification level of at least 97%. Chemicals of analytical grade were used for these individual carbonyl standards, which can react with the pure DNPH(i) to form DNPH(o) derivatives. A pure form of DNPH(i) solution was also prepared and used to induce the derivitization reaction for carbonyls. These individual chemicals were used mainly to identify each CC in the case of coelution. All solutions were stored in amber bottles to preclude any volatilization or decomposition of each carbonyl. All of these standard chemicals were stored in a refrigerator between 2 and $8^{o}$C to meet the storage condition of the manufacturer.

The procedures used for the preparation of the WS can be described as follows. The WS was prepared by mixing the PS with a suitable quantity of ACN to the final volume of 1500 μL (Table 2). The standard vials were then sealed with an aluminum crimp cap to allow the least headspace under the dark conditions. These WS were generally stable for about 8 weeks in the refrigerator. For each of all possible mobile phases considered for evaluation, a five-point calibration was conducted by injecting 20 μL of five individual WS representing the concentrations of 0.15, 0.3, 0.6, 1.2, and 2.4 μg $mL^{-1}$. To facilitate the identification of individual components in each chromatogram, the WS prepared for individual components was also tested occasionally for coeluted peaks.

The detection of CCs was completed by HPLC (Lab Alliance 500) equipped with a UV-VIS detector and dsCHROM software (ISA version) for peak integration. Because the hydrophobicitiy of the hydrazones can vary widely, the use of a gradient condition is recommended to elute CCs in a reasonable time. However, as only one pump was used to handle the mobile phase in our system, we changed the composition of mobile phases for each type of testing condition with isocratic elution. Each mobile phase for comparative experiments was hence mixed and filtered through a microglass funnel coupled with refilter paper (0.45-nm polyester membrane) to prevent any contamination. All other glass apparatuses were cleaned carefully before use to reduce the risk of contamination as well. The flow rate applied for HPLC analysis in this study was maintained at a fixed flow rate of 1.5 mL $min^{-1}$ for all experiments. The detector was kept at an optimum wavelength of 360 nm.

**TABLE 2**
**Preparation of WS Containing 15 CCs-DNPH(o)**

| Calibration Point | Dilution Factor | Mixing[a] (μL) | | Concentration ($mg\ L^{-1}$) | Amount[d] (ng) |
|---|---|---|---|---|---|
| | | PS[b] | Solvent[c] | | |
| 1 | 100 | 15 | 1485 | 0.15 | 3 |
| 2 | 50 | 30 | 1470 | 0.3 | 6 |
| 3 | 25 | 60 | 1440 | 0.6 | 12 |
| 4 | 12.5 | 120 | 1380 | 1.2 | 24 |
| 5 | 6.25 | 240 | 1260 | 2.4 | 48 |

[a] Acetonitrile is added to make a total volume of 1,500 μL.

[b] Primary standard (PS ) = 15 μg $mL^{-1}$.

[c] Acetonitrile.

[d] Amount of WS injection 20 μL.

## Evaluation of Peak Separation for a Given Mobile Phase

As one criterion to estimate the reliability of each mobile phase selected for the comparative test, the quality of peak separation can be approximated numerically by the following equation[27]:

$$R_S = \frac{2(t_2 - t_1)}{(w_1 + w_2)} \qquad (1)$$

Here, t and w are the retention time and the peak width, respectively, for the two compounds of 1 and 2. The “w” value is derived by the baseline width between two tangents drawn to the peak baseline. Eq. 1 can also be modified with a half-peak width (at the half height of peak) as follows:

$$R_S = \frac{2(t_2 - t_1)}{1.7(w_{0.5,1} + w_{0.5,2})} \qquad (2)$$

Here, $w_{0.5,1}$ and $w_{0.5,2}$ are the peak widths measured at half height for peaks 1 and 2, respectively. The measurement of half-height method for the resolution quality has been commonly employed, as this approach is more convenient than that for the baseline width[27]. In fact, in the case of complicated baseline, the use of half-height width is a practical alternative method to solve such a problem[27].

On many occasions, the simulation approach of Dolan[27] was used to evaluate the extent of resolution from all coelution cases. To evaluate such cases in a reasonable manner, the following approaches were employed[26]. In case of coelution, the second peak was allocated to assign the $R_s$ value for the given coeluted pair, while the first peak was assumed to have a symmetrical shape. As such, the $R_s$ value for a given compound was calculated by its own peak and the preceding peak. Consequently, no $R_s$ value was assigned to the first eluting compound, FA. Each of the two peaks observed in our study was thus separated theoretically and evaluated to derive their own $R_s$ values, except FA. If the separation was good enough to distinguish each peak, we assigned this optimal case as level I (i.e., ≥1.75. In the case in which two peaks with different heights occurred, the quality of their separation was verified in a stepwise manner. First, the valley height between two peaks was assessed and compared with the shorter peak height of the two. The resolution of level II was assigned so that valley height between two peaks should not be >20% of the shorter peak height ($1.5 \leq R_s < 1.75$). At last, level III was assigned for the peaks with the poorest separation in which the valley height (between two peaks) was >20% of the shorter peak height ($R_s$ < 1.5). Because of large peak tailing, those peaks with level III should be considered the lowest resolution based on peak width (or half-peak width). Overall, the levels of I and II status were used arbitrarily as acceptable resolution levels for the coeluting peaks.

## RESULTS AND DISCUSSION

### Overview of Ternary Mobile Phase Experiment

The common mobile phase in the separation of aldehydes and ketones has been the mixture of ACN:water (e.g., AW = 70:30)[26]. Hence, for each experiment, the elution condition of this AW mixture was used as the main reference for all experiments conducted subsequently with diverse mobile phases. As mentioned earlier, all of our experiments were conducted in two different stages depending on the mobile composition. As shown in Table 3A, a total of 13 experiments had been initially conducted as the preliminary stage test (experiment stage I) to examine the performance of the binary and quaternary mobile phases. This type of mobile phase was prepared by mixing water with either one (binary) or all three components of THF, IP, and MeOH (quaternary). The results of this preliminary stage test were generally not reliable enough to derive stable patterns due to either the mixed peaks (coelution) or poor resolution. In general, both binary and quaternary mixtures prepared in this study were eluted rapidly (i.e., in less than 5 min); peaks were thus drawn at a very close position or coeluted with the others. The results of stage I experiments showed that none of binary mixtures was satisfactory, while those of quaternary experiments brought out too many sets of throughputs to evaluate. It was noted, however, that the patterns for quaternary mixtures improved gradually with the increasing water content (e.g., from 5 to 10 or 20%). Due to this complication, the second stage experiments (Table 3B) were undertaken to focus on the performance of ternary mobile phases. These stage II experiments were designed to cover all possible ternary combinations between water and either two of the three target solvents (I, M, and T). For this reason, the results of stage I experiments were not evaluated further, while those tests were helpful enough to infer a suitable range of water content for the stage II experiments. Thus, the composition of water in these ternary experiments was set to constitute from as little as 20 to 40% of the total mobile phases.

### Resolution Patterns of Different Experiments

#### *Assessment of the Main Reference Mobile Phase (ACN:Water)*

For the reference mobile phase, ACN:water had been recommended by Supelco. Since then, many organizations or institutions have used such a formula as one of the optimized separation conditions for CCs-DNPH(o). Fig. 1A shows a typical chromatogram of CCs obtained by this reference mobile phase (experiment No. 100 in Tables 3A and B). The use of this mobile phase typically allowed the elution of all 15 CC-DNPH(o) within 12 min, although as many as seven out of 15 peaks were not fully separated. The resolution status of the reference experiment No. 100 is organized and shown in Table 4A. If the level of the resolution is assessed for each peak, as many as 10 compounds fell into a category of well-separated peaks in this reference chromatogram (i.e., the level of I and II). Pairs of peaks, such as IV (9) and VA (10), were resolved with the resolution level II. Note that the numbers in parenthesis denote the order in which CCs are organized in Table 1. However, except for those 10 peaks, the poorest resolution (III) was seen from the following five CCs: AR (3), OT (11), MT (12), HA (14), and DB (15). The two peak groups of (1) OT (11) and MT (12) and (2) HA (14) and DB (15) had the lowest $R_s$ value with level III. The results of Supelco showed a pattern comparable to our study, although their run time for analysis (18 min) was slightly extended relative to ours (12 min)[28]. Although Prieto-Blanco et al.[30] used shorter column (C18 Symmetry® 3.5-µm particle size [150- × 3.0-mm ID] with a precolumn 3.5 µm

**TABLE 3A**
**List of Experiments Conducted in Preliminary Stage (Exp Stage I)**

| Mixing Type | Exp No. | Mixing Phase | Mobile Phase Mixing Percentage (%) | | | | | Run Time (min)[a] |
|---|---|---|---|---|---|---|---|---|
| | | | Acetonitrile (A) —C≡N | Methanol (M) $CH_3$—OH | Water (W) $H_2O$ | Tetrahydrofuran (T)  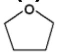 | Isopropanol (I)  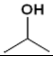 | |
| (a) Reference | 100 | AW | 70 | — | 30 | — | — | 12 |
| | 121 | MW | — | 90 | 10 | — | — | 6 |
| | 122 | MI | — | 90 | — | — | 10 | 3 |
| | 123 | MT | — | 90 | — | 10 | — | 3 |
| (b) Binary | 124 | MT | — | 80 | — | 20 | — | 3 |
| | 125 | MT | — | 70 | — | 30 | — | 3 |
| | 126 | MT | — | 60 | — | 40 | — | 3 |
| | 141 | WMTI | — | 80 | 5 | 5 | 10 | 4 |
| | 142 | WMTI | — | 80 | 10 | 5 | 5 | 5 |
| (c) Quaternary | 143 | WMTI | — | 70 | 10 | 10 | 10 | 5 |
| | 144 | WMTI | — | 60 | 10 | 10 | 20 | 5 |
| | 145 | WMTI | — | 60 | 10 | 20 | 10 | 5 |
| | 146 | WMTI | — | 60 | 20 | 10 | 10 | 5 |

[a] Column flow rate for all experiments fixed at 1.5 mL $min^{-1}$.

[10- × 2.1-mm ID]), these authors were able to determine both CCs and PAHs simultaneously in airborne particulates. The pair of AT and PA, and geometrical isomers of (o-, m-, and p-) tolualdehyde, have commonly been cited as the mixtures of poor separation[1,29]. Nonetheless, if the calibration slope values for this binary elution (ACN:water = 70:30) are assessed, the results confirm good linearities for all 15 peaks from five-point calibrations ($R^2 > 0.99$). As these compounds are determined in less than 12 min, the results of these binary mobile mixtures can be used to provide highly valuable information to check the elution conditions for all CCs determined by various mobile phases.

## *Evaluation of the Ternary Mixture Type 1 - WIM Mobile Phase*

As the first set of our ternary phase tests, the result of the WIM experiments was examined initially. The composition of the WIM phase was adjusted to vary with the 5 to 10% incremental volume of each solvent (Table 3B). In general, when the water content of experiments exceeded 30%, the HPLC system was automatically transitioned to the stand-by mode due to overpressure of the system (experiments No. 209, 210, and 211). In these cases, pump pressure was exceeding the permissible range to shut down the HPLC. Consequently, we had to ignore the results derived with all those suffering from pressure problems. In addition, the changes in IP content (e.g., IP > 20%) in conjunction with increasing water were also sensitive enough to disable the system in the same manner (e.g., experiment No. 204). Hence, these conditions were considered in the basic design of the ternary phase experiments.

Fig. 1 illustrates specifically some optimum cases of chromatograms derived by the WIM phase. As there were no significant differences in their separation patterns, these results were compared at water content levels of 20, 25, and 30%. In the case of the 20% water level, all peaks showed good resolutions except for the following five peaks: AT (4), PA (5), OT (11), MT (12), and PT (13) (Fig. 1B). As water content started to increase from 20%, the resolution patterns for several peaks (OT, MT, and PT) improved considerably. Ochs et al.[29] reported that all types of binary combination between water and MeOH are not satisfactory to separate CCs. However, they found that the addition of isopropanol can make pump pressure higher than the pure binary phase (i.e., without isopropanol), as is observed in our study. The general indications of our study result thus confirm the fact that an increase in water content facilitates the separation of peaks, although the maximum quantity of water is limited by approximately up

**TABLE 3B**
**List of All Ternary Phase Experiments (Exp Stage II)**

| Mixing Phase | Exp No. | Mobile Phase Mixing Percentage (%) | | | | | Run Time (min)[a] |
|---|---|---|---|---|---|---|---|
| | | Acetonitrile (A) —C≡N | Water (W) $H_2O$ | Isopropanol (I)  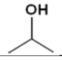 | Tetrahydrofuran (T)  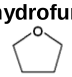 | Methanol (M) $CH_3$—OH | |
| | 100 | 70 | — | — | — | 30 | 12 |
| | 201 | — | 20 | 10 | — | 70 | 13 |
| | 202 | — | 20 | 15 | — | 65 | 11 |
| | 203 | — | 20 | 20 | — | 60 | 9 |
| | 204 | — | 20 | 30 | — | 50 | NA[b] |
| | 205 | — | 25 | 5 | — | 70 | 24 |
| (a) WIM | 206 | — | 25 | 10 | — | 65 | 21 |
| | 207 | — | 30 | 5 | — | 65 | 43 |
| | 208 | — | 30 | 10 | — | 60 | 34 |
| | 209 | — | 30 | 20 | — | 50 | NA |
| | 210 | — | 35 | 5 | — | 60 | NA |
| | 211 | — | 40 | 10 | — | 50 | NA |
| | 301 | — | 20 | — | 10 | 70 | 13 |
| | 302 | — | 20 | — | 15 | 65 | 10 |
| | 303 | — | 20 | — | 20 | 60 | 9 |
| | 304 | — | 20 | — | 30 | 50 | 7 |
| | 305 | — | 25 | — | 5 | 70 | 23 |
| | 306 | — | 25 | — | 10 | 65 | 22 |
| (b) WTM | 307 | — | 30 | — | 5 | 65 | 37 |
| | 308 | — | 30 | — | 10 | 60 | 38 |
| | 309 | — | 30 | — | 20 | 50 | 23 |
| | 310 | — | 35 | — | 5 | 60 | 86 |
| | 311 | — | 40 | — | 10 | 50 | NA |
| | 401 | — | 20 | 10 | 70 | — | 3 |
| | 402 | — | 20 | 20 | 60 | — | 3 |
| | 403 | — | 20 | 30 | 50 | — | 3 |
| | 404 | — | 20 | 40 | 40 | — | NA |
| (c) WIT | 405 | — | 25 | 10 | 65 | — | 4 |
| | 406 | — | 25 | 20 | 55 | — | 4 |
| | 407 | — | 25 | 30 | 45 | — | NA |
| | 408 | — | 30 | 10 | 60 | — | 4 |
| | 409 | — | 30 | 20 | 50 | — | NA |
| | 410 | — | 30 | 30 | 40 | — | NA |

[a] Column flow rate for all experiments fixed at 1.5 mL $min^{-1}$.

[b] NA: s.

to 35%. Note that the separation of all compounds was completed in less than 12 min for the mobile phases with the least water content (20%), while those of 30% lasted up to 35 min. As such, the highest resolution was achieved at or near the maximum content of water (30%), despite the counteractive extension of run time. In addition, as the extended run time consumes more mobile phases, one must find the compensation point between the numerous contrasting aspects.

## *Evaluation of the Ternary Mixture Type 2 - WTM Mobile Phase*

As the second set of ternary phase experiments, all matches for WTM were examined at 5% incremental volume of water from 20 to 35% (Table 3B). In most experiments done in the WTM phase, carbonyls were separated fairly effectively with the exception of experiment No. 311. This extraordinary pattern in experiment No. 311 is due to excess water (e.g., 40%), which caused an overpressure problem. Note that

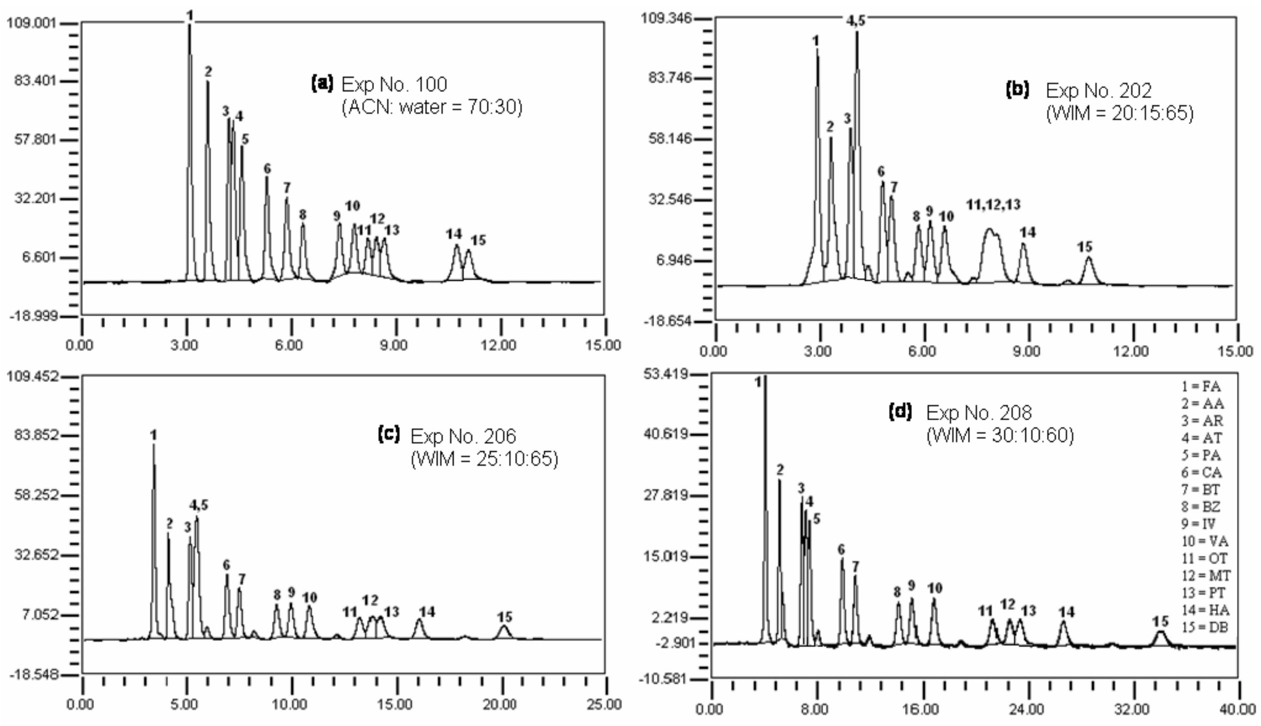

**FIGURE 1.** Chromatogram of reference experiment (No. 100; ACN:water = 70:30) and three WIM phase experiments with working standards (48 ng of CCs).

even at 35% water (experiment No. 310), the system was not able to operate properly due to excessive pump pressure. As shown in Fig. 2, tailings of coeluted peaks, e.g., MT (12), and PT (13), were observed in many WTM experiments. Coelution of a peak group, AR (3) and AT (4), tends to occur consistently in experiments with water content of 20 and 25% (e.g., experiments No. 301 and 305). In most WTM experiments, the separation of three compounds, OT (11), MT (12), and MT (13), was generally restricted except for experiment No. 310. These three compounds eluted either as a mixed peak (experiments No. 301–304) or with poor separation (e.g., experiments No. 305–309). Although many modern HPLC methods rely on the gradient mobile phase as an eluent for aldehyde/ketone analysis, tolualdehyde with three different isomers (o-, m-, and p-) is still recorded as the most unseparable group due to the same polarity[30]. These authors found that the addition of THF in the ternary mobile phase at low percentage led to a better resolution for the lighter CCs across AR, AT, and PA. It is interesting to note that they were able to separate AT from AR using a column (Symnetry 150 mm) that is shorter than ours (Hichrom 250 mm).

## Evaluation of the Ternary Mixture Type 3 - WIT Mobile Phase

As the third set of the ternary mobile phase, the patterns for WIT phase were evaluated (Table 3). All WIT experiments were conducted in a manner analogous to those of the WIM and WTM phases. The results of WIT phases organized at incremental volume of water from 5 or 10% show that four out of 10 combinations (e.g., experiments No. 404, 407, 409, and 410) did not work properly due to an excessive pressure problem, as explained above. This overpressure problem occurred very frequently from the WIT phases relative to other ternary phases, as shown in each type of water composition (Fig. 3). It is thus expected that the increase in water content of the WIT mobile phase should promote the overpressure

**TABLE 4**
**Results of all $R_s$ Values for All Experiments Conducted in Experiment Stage 2 (Ternary Mobile Phases)**

A. Reference Mobile Phase of Acetonitrile:Water (AW = 70:30) Experiment

| Order | | 1 | 2 | 3 | 4 | 5 | 6 | 7 | 8 | 9 | 10 | 11 | 12 | 13 | 14 | 15 |
|---|---|---|---|---|---|---|---|---|---|---|---|---|---|---|---|---|
| **Compound** | | **FA** | **AA** | **AR** | **AT** | **PA** | **CA** | **BT** | **BZ** | **IV** | **VA** | **OT** | **MT** | **PT** | **HA** | **DB** |
| Exp No.100 | Slope | 325,647 | 250,084 | 249,194 | 219,493 | 208,441 | 162,054 | 148,622 | 103,194 | 96,831 | 74,658 | 35,200 | 16,849 | 24,083 | 23,138 | 18,010 |
| 70:30 | $R_s$ | NA | 3.14 (I) | 4.58 (I) | 1.22 (III) | 1.73 (II) | 3.68 (I) | 2.52 (I) | 1.91 (I) | 4.27 (I) | 1.57 (II) | 1.72 (II) | 1.32 (III) | 1.17 (III) | 8.83 (I) | 1.19 (III) |

B. Ternary Mobile Phase of Water:Isohydrofuran:Methanol (WIM) Experiments

| Order | | 1 | 2 | 3 | 4 | 5 | 6 | 7 | 8 | 9 | 10 | 11 | 12 | 13 | 14 | 15 |
|---|---|---|---|---|---|---|---|---|---|---|---|---|---|---|---|---|
| **Compound** | | **FA** | **AA** | **AR** | **AT** | **PA** | **CA** | **BT** | **BZ** | **IV** | **VA** | **OT** | **MT** | **PT** | **HA** | **DB** |
| | | | | | **AT+PA** | | | | | | | **OT+MT+PT** | | | | |
| Exp No.201 | Slope[a] | 298,875 | 220,188 | 76,261 | 206,287 | | 127,564 | 98,710 | 85,547 | 92,233 | 114,544 | 269,192 | | | 87,257 | 82,162 |
| 20:10:70 | $R_s$[b] | NA[c] | 2.02 (I) | 3.11 (I) | 1.18 (III) | | 3.95 (I) | 1.24 (III) | 3.57 (I) | 1.24 (III) | 1.53 (II) | 2.17 (I) | | | 1.41 (III) | 5.26 (I) |
| | | | | | **AT+PA** | | | | | | | **OT+MT** | | | | |
| Exp No.202 | Slope | 314,729 | 227,660 | 82,908 | 236,996 | | 119,327 | 96,683 | 86,737 | 92,535 | 89,813 | 62,424 | | 20,696 | 101,967 | 88,565 |
| 20:15:65 | $R_s$ | NA | 1.99 (I) | 3.34 (I) | 1.2 (III) | | 3.71 (I) | 1.2 (III) | 3.16 (I) | 1.22 (III) | 1.4 (III) | 2.75 (I) | | 0.41 (III) | 1.68 (II) | 4.37 (I) |
| | | | | | **AT+PA** | | | | | | | **OT+MT+PT** | | | | |
| Exp No.203 | Slope | 301,039 | 201,281 | 66,516 | 200,323 | | 105,113 | 85,486 | 89,886 | 82,795 | 80,627 | 279,506 | | | 87,026 | 58,171 |
| 20:20:60 | $R_s$ | NA | 1.82 (I) | 3.18 (I) | 1.18 (III) | | 3.56 (I) | 2.07 (I) | 4.43 (I) | 1.2 (III) | 1.33 (III) | 2.03 (I) | | | 1.52 (II) | 3.92 (I) |
| | | | | | **AT+PA** | | | | | | | | | | | |
| Exp No.205 | Slope | 299,713 | 225,713 | 115,394 | 211,206 | | 142,278 | 111,013 | 84,118 | 93,124 | 106,353 | 56,915 | 26,249 | 29,925 | 77,427 | 62,220 |
| 25:5:70 | $R_s$ | NA | 3.12 (I) | 4.66 (I) | 1.11 (III) | | 3.98 (I) | 2.25 (I) | 4.64 (I) | 1.54 (II) | 2.16 (I) | 5.9 (I) | 1.59 (II) | 1.27 (III) | 4.01 (I) | 7.3 (I) |
| | | | | | **AT+PA** | | | | | | | | | | | |
| Exp No.206 | Slope | 320,593 | 240,765 | 113,413 | 222,642 | | 150,001 | 117,777 | 97,357 | 99,468 | 115,571 | 55,615 | 24,610 | 27,053 | 85,276 | 73,813 |
| 25:10:65 | $R_s$ | NA | 2.83 (I) | 4.46 (I) | 1.15 (III) | | 4.19 (I) | 1.69 (II) | 4.43 (I) | 1.59 (II) | 1.87 (I) | 5.13 (I) | 1.57 (II) | 1.31 (III) | 4.21 (I) | 7.68 (I) |
| Exp No.207 | Slope | 384,612 | 151,998 | 131,233 | 81,987 | 120,635 | 187,268 | 148,480 | 111,682 | 118,695 | 139,701 | 95,206 | 62,941 | 63,891 | 104,446 | 85,150 |
| 30:5:65 | $R_s$ | NA | 4.87 (I) | 7.72 (I) | 1.17 (III) | 1.26 (III) | 7.21 (I) | 2.29 (I) | 7.28 (I) | 1.58 (II) | 2.82 (I) | 7.37 (I) | 2.06 (I) | 1.4 (III) | 4.29 (I) | 8.84 (I) |
| Exp No.208 | Slope | 376,456 | 219,309 | 229,909 | 212,219 | 207,840 | 183,725 | 146,023 | 114,252 | 122,446 | 138,187 | 84,156 | 49,923 | 50,504 | 97,447 | 76,268 |
| 30:10:60 | $R_s$ | NA | 4.57 (I) | 7.23 (I) | 1.22 (III) | 1.18 (III) | 7.07 (I) | 2.07 (I) | 6.14 (I) | 1.73 (II) | 2.55 (I) | 6.56 (I) | 2.08 (I) | 1.52 (II) | 4.64 (I) | 7.81 (I) |

[a] Slope value derived from five-point calibration of WS.

[b] Peak resolution value presents peak separation and roman in parenthesis symbolizes for separation level.

[c] NA: not applicable.

*Table 4 continues next page.*

problem of the HPLC system. As all WIT experiments were subject to the highest pressure conditions, most peaks exhibited very poor separation with the least number of peaks (4–7) (Fig. 3). As a result, the run time for most WIT experiments was fairly short (e.g., <4 min) with many cases of coelution. Overall, these WIT experiments were not effective in yielding good separation of CCs. Due to this restriction, we confined most of our discussions to experimental results derived from the WIM and WTM phases.

## The Effect of Three Key Variables in HPLC Analysis

### *Comparison of $R_s$ between Different Ternary Phases*

In order to allow the comparison of peak separation patterns between different ternary mobile phases, $R_s$ values derived for each individual mobile phase composition are documented and compared in Table 4. The results of both WIM and WTM experiments indicate that the poorest resolutions tend to occur most intensively in either lighter carbonyls of AR, AT, and PA (3–5) or heavier ones of OT, MT, and PT (11–13). The distinction of resolution between aliphatic, aromatic, and unsaturated CCs were also observed[31]. The increase of carbon chain in molecular structure was attributed to the main cause of poor separation (e.g., the formation of geometrical isomers between OT, MT, and PT).

## TABLE 4 (continued)
## Results of all $R_s$ Values for All Experiments Conducted in Experiment Stage 2 (Ternary Mobile Phases)

C. Ternary Mobile Phase of Water:Methanol:Tetrahydrofuran (WTM) Experiments

| Order | | 1 | 2 | 3 | 4 | 5 | 6 | 7 | 8 | 9 | 10 | 11 | 12 | 13 | 14 | 15 |
|---|---|---|---|---|---|---|---|---|---|---|---|---|---|---|---|---|
| **Compound** | | **FA** | **AA** | **AR** | **AT** | **PA** | **CA** | **BT** | **BZ** | **IV** | **VA** | **OT** | **MT** | **PT** | **HA** | **DB** |
| | | | | **AR+AT** | | | | | | | | | **OT+MT+PT** | | | |
| Exp No.301 | Slope[a] | 281,769 | 226,110 | 243,470 | | 51,328 | 145,882 | 126,260 | 92,373 | 77,577 | 71,339 | | 279,788 | | 80,834 | 64,474 |
| 20:10:70 | $R_s$[b] | NA[c] | 1.98 (I) | 2.72 (I) | | 0.69 (III) | 2.94 (I) | 1.22 (III) | 2.63 (I) | 1.54 (II) | 1.39 (III) | | 3.17 (I) | | 2.73 (I) | 4.10 (I) |
| | | | | **AR+AT** | | | | | | | | | **OT+MT+PT** | | | |
| Exp No.302 | Slope | 279,417 | 207,375 | 200,730 | | 32,153 | 132,828 | 120,827 | 93,919 | 73,670 | 70,540 | | 250,490 | | 71,517 | 74,769 |
| 20:15:65 | $R_s$ | NA | 1.77 (I) | 2.6 (I) | | 0.53 (III) | 2.11 (I) | 1.23 (III) | 1.93 (I) | 1.75 (I) | 1.17 (III) | | 2.7 (I) | | 3.2 (I) | 3.09 (I) |
| | | | | **AR+AT** | | | | | | | | | **OT+MT+PT** | | | |
| Exp No.303 | Slope | 248,871 | 192,260 | 606,463 | | | 124,668 | 111,399 | 86,329 | 58,349 | 54,934 | | 274,103 | | 105,814 | 68,150 |
| 20:20:60 | $R_s$ | NA | 1.62 (II) | 1.51 (II) | | | 1.99 (I) | 1.22 (III) | 1.56 (II) | 1.96 (I) | 1.19 (III) | | 2.17 (I) | | 2.91 (I) | 2.17 (I) |
| | | | | **AR+AT** | | | | | | | | | **OT+MT+PT** | | | |
| Exp No.304 | Slope | 242,067 | 183,704 | 550,967 | | | 114,571 | 77,002 | 48,271 | 38,636 | 26,568 | | 187,374 | | 70,319 | 43,577 |
| 20:30:50 | $R_s$ | NA | 1.35 (III) | 1.28 (III) | | | 1.48 (III) | 1.29 (III) | 1.18 (III) | 3.44 (I) | 1.24 (III) | | 1.37 (III) | | 3.65 (I) | 1.24 (III) |
| | | | | **AR+AT** | | | | | | | | | **MT+PT** | | | |
| Exp No.305 | Slope | 365,201 | 273,371 | 363,534 | | 133,569 | 175,718 | 113,724 | 111,716 | 102,275 | 90,071 | 32,135 | 99,849 | | 91,446 | 79,949 |
| 25:5:70 | $R_s$ | NA | 2.91 (I) | 4.01 (I) | | 1.21 (III) | 4.91 (I) | 1.91 (I) | 5.11 (I) | 1.72 (II) | 2.15 (I) | 5.74 (I) | 0.75 (III) | | 3.18 (I) | 6.93 (I) |
| | | | | **AR+AT** | | | | | | | | | **OT+MT+PT** | | | |
| Exp No.306 | Slope | 354,256 | 248,400 | 351,444 | | 140,366 | 171,313 | 151,589 | 111,669 | 122,186 | 86,282 | | 284,446 | | 80,631 | 79,668 |
| 25:10:65 | $R_s$ | NA | 2.76 (I) | 3.81 (I) | | 1.19 (III) | 4.25 (I) | 1.72 (II) | 3.9 (I) | 1.82 (I) | 1.73 (II) | | 3.76 (I) | | 3.26 (I) | 5.36 (I) |
| | | | | **AR+AT** | | | | | | | | | | | | |
| Exp No.307 | Slope | 414,291 | 305,818 | 502,717 | | 219,825 | 209,080 | 127,220 | 129,368 | 110,513 | 121,237 | 86,671 | 29,411 | 30,492 | 105,745 | |
| 30:5:65 | $R_s$ | NA | 4.16 (I) | 5.49 (I) | | 1.59 (II) | 6.82 (I) | 2.81 (I) | 8.47 (I) | 2.01 (I) | 3.1 (I) | 7.76 (I) | 1.16 (III) | 0.53 (III) | 4.65 (I) | |
| | | | | | | | | | | | | | **MT+PT** | | | |
| Exp No.308 | Slope | 317,978 | 257,676 | 167,490 | 169,624 | 138,478 | 136,379 | 130,703 | 86,528 | 113,507 | 104,479 | 30,720 | 90,565 | | 105,417 | 77,054 |
| 30:10:60 | $R_s$ | NA | 3.64 (I) | 6.3 (I) | 1.2 (III) | 1.57 (II) | 5.74 (I) | 2.33 (I) | 5.98 (I) | 2.02 (I) | 2.11 (I) | 8.55 (I) | 1.23 (III) | | 5.76 (I) | 6.88 (I) |
| | | | | | | | | | | | | | **OT+MT+PT** | | | |
| Exp No.309 | Slope | 321,526 | 228,170 | 139,857 | 101,349 | 88,995 | 148,129 | 140,739 | 96,943 | 109,297 | 109,521 | | 246,150 | | 100,046 | 65,972 |
| 30:20:50 | $R_s$ | NA | 2.59 (I) | 3.59 (I) | 1.42 (III) | 1.16 (III) | 4.76 (I) | 2.02 (I) | 3.54 (I) | 2.59 (I) | 1.46 (III) | | 3.49 (I) | | 5.35 (I) | 3.39 (I) |
| Exp No.310 | Slope | 306,391 | 227,312 | 155,913 | 208,132 | 165,790 | 146,165 | 135,194 | 85,393 | 111,189 | 77,999 | 69,353 | 23,638 | 27,944 | 69,010 | 73,027 |
| 35:5:65 | $R_s$ | NA | 5.59 (I) | 10.88 (I) | 1.25 (III) | 2.51 (I) | 8.58 (I) | 3.33 (I) | 10.16 (I) | 1.88 (I) | 3.9 (I) | 10.66 (I) | 1.74 (II) | 0.59 (III) | 5.43 (I) | 12.81 (I) |

[a] Slope value derived from five-point calibration of WS.

[b] Peak resolution value presents peak separation and roman in parenthesis symbolizes for separation level.

[c] NA: not applicable.

To specifically describe the resolution trends of different CCs between WIM and WTM, the most representative $R_s$ values for the ternary phase experiments (e.g., No. 208 and 310) were normalized by the reference calibration results of ACN:water (70:30). The results of this normalization, as shown in Fig. 4, indicate that there are slight differences in resolution trends between WIM and WTM. In general, most $R_s$ values of the WTM experiments tend to maintain better resolution than WIM experiments. The only exceptions from such trends were seen from MT and PT with the least resolution level. As mentioned before, AT (4), PA (5), MT (12), and PT (13) still exhibited the poorest resolution relative to the reference experiment (No. 100).

In the case of the lighter CCs, the weakest resolution of WIM phase is observed from PA (5), while that for WTM is AT (4). The WIM results show that the $R_s$ values for PA were derived from only two out of all seven experiments, although those scarcely derived $R_s$ values tend to be significantly large enough to exhibit good resolution. In contrast, the results of WTM experiments indicate that the $R_s$ values of AT are derived from three out of all 10 experiments. Likewise, this type of contrasting trend between two mobile phases is also observed from heavier species such as MT (12) and PT (13), as summarized in Table 5. If the resolution data are evaluated in terms of maximum number of CCs with the $R_s$ rating, the experiments conducted at the highest water content are found consistently to yield the optimal patterns. Out of 10 WTM experiments, experiment No. 310 was able to yield $R_s$ values for all 15 compounds, as well as the results of experiments No. 207 and 208 in WIM phases for all 15 CCs. Hence, if one attempts

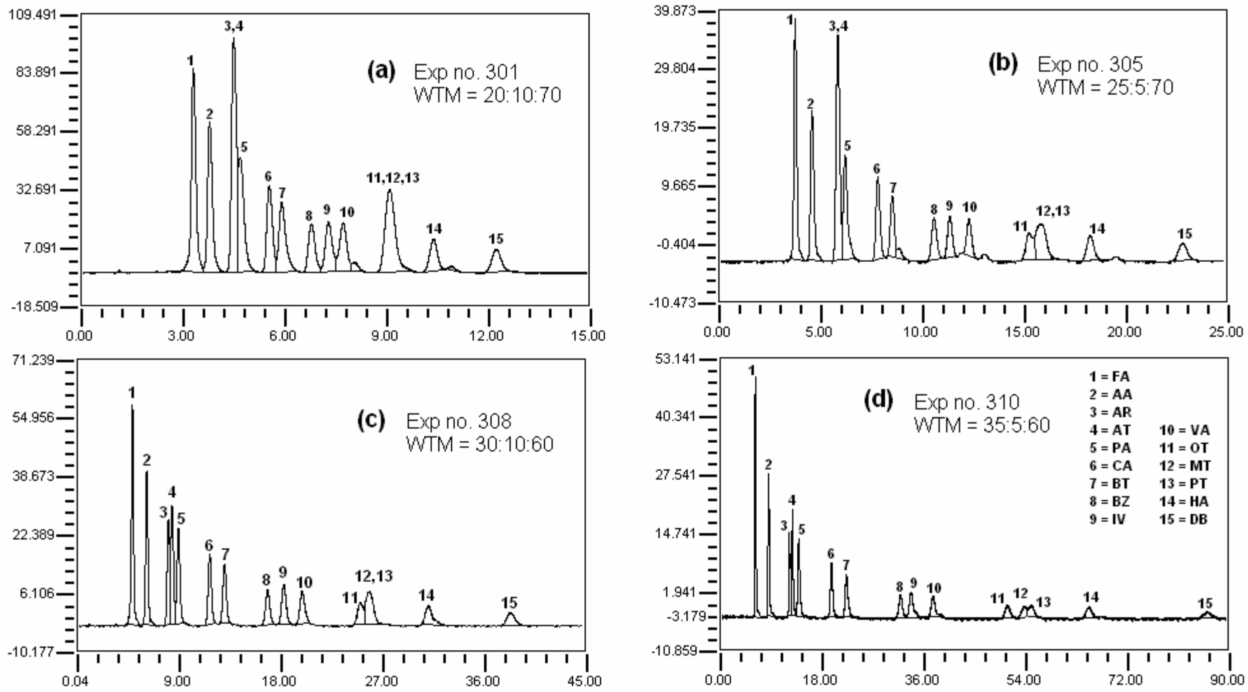

**FIGURE 2**. Chromatogram of a mixture of 15 DNPH(o) derivatives (48 ng of CCs) in WTM ternary mobile phase.

to obtain the maximum number of separation, mobile composition used in those experiments should be considered first for both mobile phases.

## Relative Sensitivity

In order to examine the relative performance of each ternary phase, the results derived from each experiment can also be evaluated on the basis of such variables as sensitivity or retention time. Note that our peak areas were integrated electronically by software (DsChrom). However, as each component in multiple peaks cannot be integrated independently, manual integration often became inevitable. To evaluate the relative ordering of sensitivity for each ternary phase combination, all calibration slope values of each CC derived from the model experiments of WIM and WTM phases were compared with those derived from ACN:water datasets. The results depicted in Fig. 4 generally indicate the enhancement of slope values with the increasing molecular weights. Although the WTM experiments for most CCs showed better $R_s$ values than those of WIM, the relative sensitivity of WTM tended to be lower than their WIM counterpart. In addition, the sensitivity of both WIM and WTM experiments is greatly distinguished between lighter and heavier CCs. Most noticeably, the sensitivity of the lighter CCs in both ternary phases tends to be moderately reduced relative to the ACN:water mixture. In contrast, the results derived from heavier CCs show significantly enhanced sensitivity in both phases. One can thus conclude that the use of these ternary mobile phases should affect the sensitivity of the HPLC technique in relation to the molecular weight of the target CCs and type of mobile phase combination, to a certain extent.

## Retention Time

In general, the better resolution of multiple peaks often requires an extension of the run time for allowing each peak to appear at its own retention time. Hence, it is necessary to find a balancing point that can satisfy

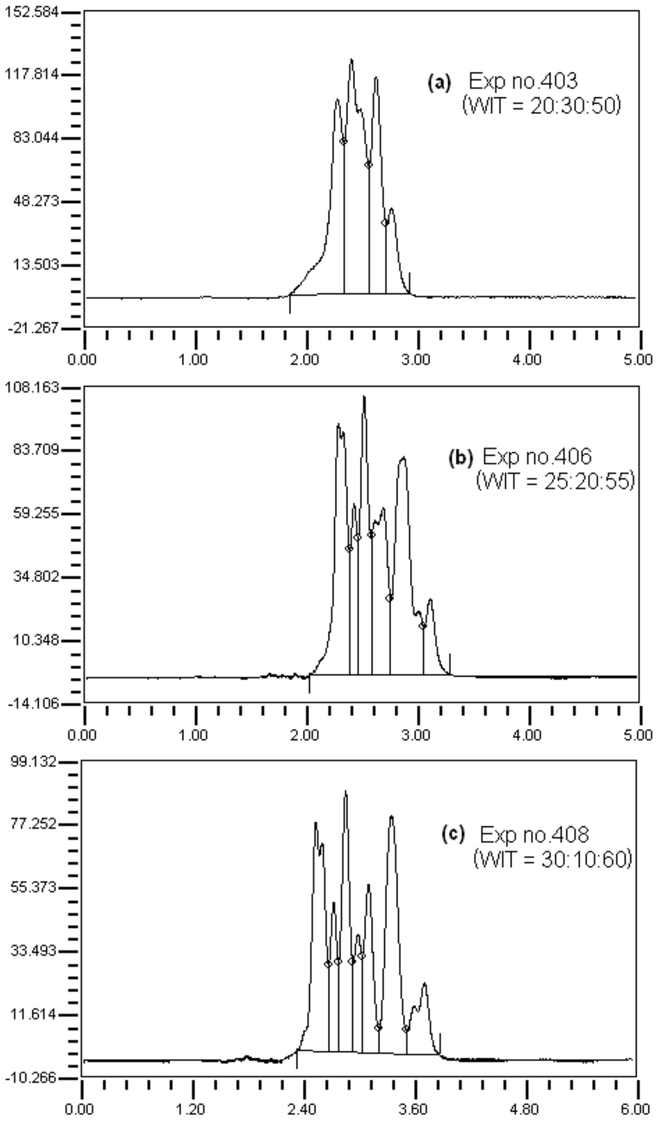

**FIGURE 3.** Chromatogram of a mixture of 15 DNPH(o) derivatives (48 ng of CCs) in WIT ternary mobile phase with detection at 360 nm.

good resolution within a reasonably short run time. To assess the basic properties of separation by ternary phases investigated in this study, the run times of all CCs are summarized in Table 3. As an important parameter in the qualitative analysis, information of this run time data provides not only time of elution, but also the volume of mobile phase for consumption. The results of stage I experiments generally showed fairly short run times for both binary and quaternary (e.g., <5 min). In the case of experiments done with ternary mobile phases (experiment stage II), such fast separation was also seen in the WIT phase. In contrast, the results of the other two ternary mobile phases (WIM and WTM) were recorded in a greatly extended time for the completion of each experiment. The run time values of these latter two ternary phases varied in a wide range from 12 to 90 min as a function of their relative composition. The longer run time accompanied by the increased water content was also observed in a previous study (e.g., [32]). Besides, run time is known to be affected by other factors, such as temperature, pH value of mobile phase, elutent, or the kind of eluting column[14,20].

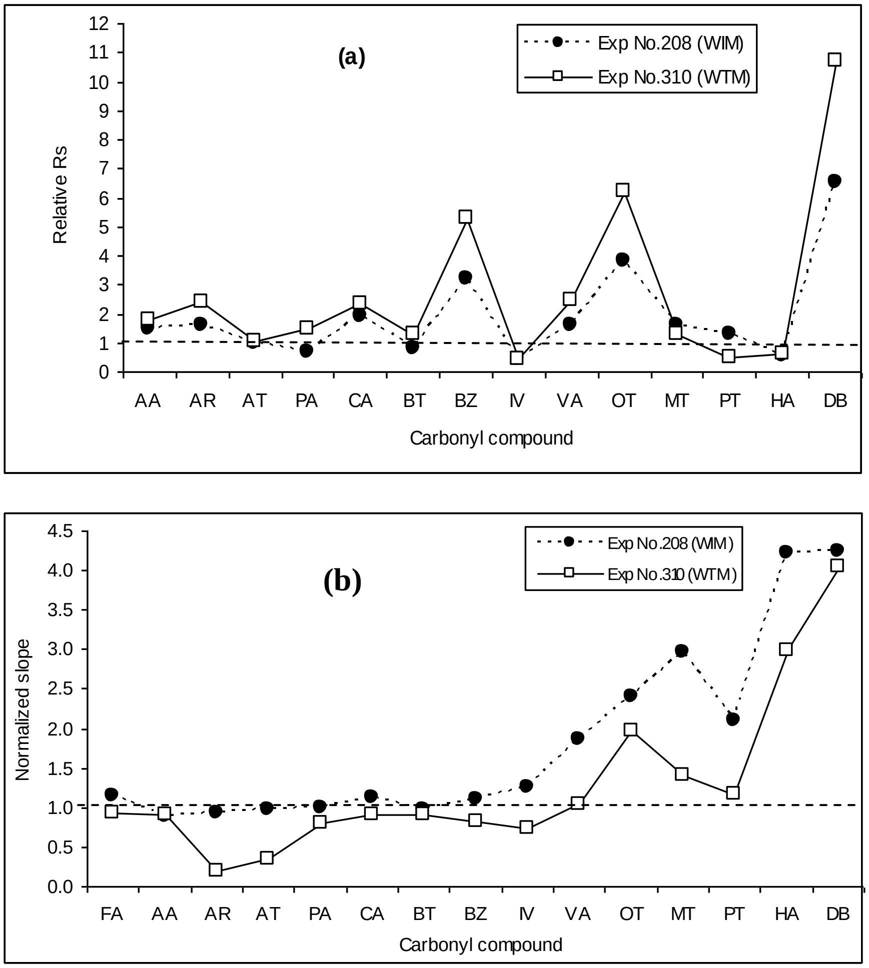

**FIGURE 4.** Normalization of experimentally determined resolution values ($R_S$) and slope values: WIM and WTM phases, relative to ACN:water (70:30) phase.

In our study, water composition in each combination was consistently found as a key variable to the determination of run time patterns. At low water content (≤20%), CCs were eluted rapidly from the column (~10 min). However, significant increases in run time were accompanied by increased water content (e.g., 30 and 35%) (Table 5). Because of the effect of water content on run time, the best resolution (or maximum separation) of CCs was attained with the ternary phases with the increasing water content (e.g., ≥30%).

## The Use of Methanol/Water (M/W) Ratio to Evaluate the Relative Performance of Key Parameters

To learn more about the relative importance of different parameters on a parallel basis, we applied the M/W ratio to assess relative performance between different mixing conditions. The use of this ratio was considered because water content is a sensitive component of the analytical trend, and methanol is the dominant component of the ternary phase. Hence, all the results expressed in relation to the three variables (i.e., resolution, sensitivity, and run time) can be reorganized to allow a direct comparison against the M/W ratio (Table 6).

**TABLE 5**
**Summary of All Experiments in Terms of Resolution ($R_s$) Values between Different Experiment Modes and Compound Types**

A. Comparison between Different Experiment Modes

| Mixing Phase | Exp No. | Mobile Phase | | $R_s$ Value | | | No. of Peak | Run Time (min) |
|---|---|---|---|---|---|---|---|---|
| | | Composition | Mean | SD | Min | Max | | |
| | 201 | 20:10:70 | 2.43 | 1.36 | 1.18 | 5.26 | 13 | 12 |
| | 202 | 20:15:65 | 2.20 | 1.23 | 0.41 | 4.37 | 13 | 11 |
| | 203 | 20:20:60 | 2.39 | 1.18 | 1.18 | 4.43 | 12 | 9 |
| (a) WIM | 205 | 25:5:70 | 3.35 | 1.93 | 1.11 | 7.30 | 14 | 24 |
| | 204 | 25:10:65 | 3.24 | 1.96 | 1.15 | 7.68 | 14 | 21 |
| | 207 | 30:5:65 | 4.30 | 2.85 | 1.17 | 8.84 | 15 | 43 |
| | 208 | 30:10:60 | 4.03 | 2.52 | 1.18 | 7.81 | 15 | 34 |
| | 301 | 20:10:70 | 2.28 | 1.01 | 0.69 | 4.10 | 12 | 12 |
| | 302 | 20:15:65 | 2.01 | 0.84 | 0.53 | 3.20 | 12 | 10 |
| | 303 | 20:20:60 | 1.83 | 0.52 | 1.19 | 2.91 | 11 | 9 |
| | 304 | 20:30:50 | 1.75 | 0.95 | 1.18 | 3.65 | 11 | 7 |
| (b) WTM | 305 | 25:5:70 | 3.38 | 1.96 | 0.75 | 6.93 | 13 | 13 |
| | 306 | 25:10:65 | 3.05 | 1.31 | 1.19 | 5.36 | 12 | 22 |
| | 307 | 30:5:65 | 4.05 | 2.64 | 0.53 | 8.47 | 13 | 37 |
| | 308 | 30:10:60 | 4.10 | 2.52 | 1.20 | 8.55 | 14 | 38 |
| | 309 | 30:20:50 | 2.95 | 1.32 | 1.16 | 5.35 | 13 | 23 |
| | 310 | 35:5:65 | 5.66 | 4.16 | 0.59 | 12.81 | 15 | 86 |
| (c) WIT | — | — | — | — | — | — | — | — |

B. Comparison between Different Compounds

| CC | $R_s$ of WIM phase ($n_{max}$ =7) | | | | | $R_s$ of WTM phase ($n_{max}$ = 10) | | | | |
|---|---|---|---|---|---|---|---|---|---|---|
| | Mean | SD | Min | Max | N | Mean | SD | Min | Max | N |
| FA | NA * | NA | NA | NA | NA | NA | NA | NA | NA | NA |
| AA | 3.03 | 1.25 | 1.82 | 4.87 | 7 | 2.84 | 1.32 | 1.35 | 5.59 | 10 |
| AR | 4.81 | 1.92 | 3.11 | 7.72 | 7 | 4.22 | 2.82 | 1.28 | 10.88 | 10 |
| AT | 1.17 | 0.04 | 1.11 | 1.22 | 7 | 1.29 | 0.11 | 1.20 | 1.42 | 3 |
| PA | 1.22 | 0.06 | 1.18 | 1.26 | 2 | 1.31 | 0.61 | 0.53 | 2.51 | 8 |
| CA | 4.81 | 1.60 | 3.56 | 7.21 | 7 | 4.36 | 2.29 | 1.48 | 8.58 | 10 |
| BT | 1.83 | 0.46 | 1.20 | 2.29 | 7 | 1.91 | 0.73 | 1.22 | 3.33 | 10 |
| BZ | 4.81 | 1.44 | 3.16 | 7.28 | 7 | 4.45 | 3.01 | 1.18 | 10.16 | 10 |
| IV | 1.44 | 0.22 | 1.20 | 1.73 | 7 | 2.07 | 0.55 | 1.54 | 3.44 | 10 |
| VA | 1.95 | 0.58 | 1.33 | 2.82 | 7 | 1.94 | 0.91 | 1.17 | 3.90 | 10 |
| OT | 4.56 | 2.21 | 2.03 | 7.37 | 7 | 4.94 | 3.10 | 1.37 | 10.66 | 10 |
| MT | 1.83 | 0.29 | 1.57 | 2.08 | 4 | 1.22 | 0.40 | 0.75 | 1.74 | 4 |
| PT | 1.18 | 0.44 | 0.41 | 1.52 | 5 | 0.56 | 0.04 | 0.53 | 0.59 | 2 |
| HA | 3.11 | 1.49 | 1.41 | 4.64 | 7 | 4.01 | 1.16 | 2.73 | 5.76 | 10 |
| DB | 6.46 | 1.91 | 3.92 | 8.84 | 7 | 5.11 | 3.49 | 1.24 | 12.81 | 9 |

NA, not applicable.

**TABLE 6**
**Results of Correlation Analysis between M/W Ratio for All 15 CCs Obtained in Three Types of Data ($R_s$ Value, Slope Value, and Run Time)**

A. M/W Ratio vs. $R_s$ Value

| Compound | WIM Phase Slope | WIM Phase Offset | WIM Phase $r^2$ | N[a] | P[b] | WTM Phase Slope | WTM Phase Offset | WTM Phase $r^2$ | N | P |
|---|---|---|---|---|---|---|---|---|---|---|
| FA | NA | NA | NA | NA | NA | NA | NA | NA | NA | NA |
| AA | -2.090 | 8.793 | 0.841 | 7 | 3.7E-03 | -1.538 | 6.713 | 0.541 | 10 | 4.3E-03 |
| AR | -3.256 | 13.791 | 0.864 | 7 | 2.5E-03 | -3.112 | 12.063 | 0.483 | 10 | 2.1E-02 |
| AT | -0.012 | 1.205 | 0.035 | 7 | 8.2E-01 | -0.019 | 1.336 | 0.569 | 3 | 9.3E-01 |
| PA | 0.008 | 1.199 | 1.000 | 2 | — | -0.691 | 3.048 | 0.608 | 8 | 1.8E-02 |
| CA | -2.516 | 11.747 | 0.740 | 7 | 1.3E-02 | -2.865 | 11.577 | 0.622 | 10 | 6.8E-03 |
| BT | -0.612 | 3.516 | 0.531 | 7 | 6.3E-02 | -0.963 | 4.335 | 0.684 | 10 | 4.8E-02 |
| BZ | -2.345 | 11.278 | 0.798 | 7 | 6.8E-03 | -3.323 | 12.821 | 0.486 | 10 | 2.8E-01 |
| IV | -0.352 | 2.414 | 0.798 | 7 | 6.5E-03 | -0.194 | 2.561 | 0.048 | 10 | 7.0E-02 |
| VA | -0.927 | 4.508 | 0.769 | 7 | 9.6E-03 | -0.922 | 4.266 | 0.407 | 10 | 6.0E-02 |
| OT | -3.593 | 14.467 | 0.794 | 7 | 7.1E-03 | -3.308 | 13.272 | 0.454 | 10 | 4.4E-02 |
| MT | -0.314 | 2.692 | 0.902 | 4 | 4.8E-02 | -0.417 | 2.271 | 0.955 | 4 | — |
| PT | -0.657 | 2.993 | 0.782 | 5 | 4.6E-02 | -0.017 | 0.608 | 1.000 | 2 | — |
| HA | -2.415 | 9.767 | 0.795 | 7 | 7.0E-03 | -1.660 | 8.196 | 0.811 | 10 | 1.7E-01 |
| DB | -2.843 | 14.298 | 0.666 | 7 | 2.5E-02 | -3.029 | 12.740 | 0.327 | 9 | 1.3E-01 |

B. M/W Ratio vs. Slope Value

| Compound | Slope | Offset | $r^2$ | N[a] | P[b] | Slope | Offset | $r^2$ | N | P |
|---|---|---|---|---|---|---|---|---|---|---|
| FA | -58203 | 488614 | 0.748 | 7 | 1.2E-02 | -29847 | 388384 | 0.122 | 10 | 3.2E-01 |
| AA | 20617 | 155524 | 0.150 | 7 | 3.9E-01 | -17128 | 278177 | 0.084 | 10 | 4.2E-01 |
| AR | -84741 | 359364 | 0.706 | 7 | 1.8E-02 | 86358 | 4806 | 0.083 | 4 | 8.0E-03 |
| AT | -781392 | 2.E+06 | 1.000 | 2 | — | 52648 | 59792 | 0.984 | 3 | 8.3E-01 |
| PA | -523228 | 1.E+06 | 1.000 | 2 | — | -54365 | 255170 | 0.372 | 8 | 1.1E-01 |
| CA | -50204 | 283578 | 0.773 | 7 | 9.1E-03 | -7880 | 170328 | 0.032 | 10 | 6.3E-01 |
| BT | -40041 | 225376 | 0.807 | 7 | 6.0E-03 | -9440 | 147251 | 0.086 | 10 | 4.1E-01 |
| BZ | -20870 | 153246 | 0.823 | 7 | 4.8E-03 | -624 | 95822 | 3.E-04 | 10 | 9.6E-01 |
| IV | -23236 | 164306 | 0.741 | 7 | 1.3E-02 | -23804 | 151702 | 0.289 | 10 | 1.1E-01 |
| VA | -29772 | 194271 | 0.536 | 7 | 6.2E-02 | -20636 | 133296 | 0.220 | 10 | 1.7E-01 |
| OT | -47216 | 185898 | 0.784 | 4 | 1.2E-01 | -26715 | 112697 | 0.195 | 4 | 5.6E-01 |
| MT | -43146 | 144123 | 0.736 | 4 | 1.4E-01 | 12761 | 1762 | 1.000 | 2 | — |
| PT | -30397 | 118895 | 0.755 | 5 | 5.7E-02 | 5633 | 18288 | 1.000 | 2 | — |
| HA | -6022 | 108167 | 0.111 | 7 | 4.7E-01 | -5607 | 102206 | 0.053 | 10 | 5.3E-01 |
| DB | 334 | 74272 | 3.E-04 | 7 | 9.7E-01 | -370 | 70572 | 5.E-04 | 10 | 8.7E-01 |

[a] Number of input value pairs for correlation analysis using SPSS program.

[b] Probability: correlation is significant at the 0.05 level (two-tailed).

*Table 6 continues next page.*

**TABLE 6 (continued)**
**Results of Correlation Analysis between M/W Ratio for All 15 CCs Obtained in Three Types of Data ($R_s$ Value, Slope Value, and Run Time)**

C. M/W Ratio vs. Run Time

| Compound | Slope | Offset | $r^2$ | N[a] | P[b] | Slope | Offset | $r^2$ | N | P |
|---|---|---|---|---|---|---|---|---|---|---|
| FA | -0.897 | 5.957 | 0.772 | 7 | 9.E-03 | -1.357 | 7.546 | 0.634 | 10 | 6.0E-03 |
| AA | -1.516 | 8.398 | 0.793 | 7 | 7.E-03 | -2.076 | 10.258 | 0.582 | 10 | 1.0E-02 |
| AR | -2.501 | 12.220 | 0.799 | 7 | 7.E-03 | -3.217 | 14.532 | 0.541 | 10 | 1.5E-02 |
| AT | -2.591 | 12.739 | 0.792 | 7 | 7.E-03 | -3.443 | 15.211 | 0.572 | 10 | 1.1E-02 |
| PA | -2.828 | 13.485 | 0.797 | 7 | 7.E-03 | -3.817 | 16.546 | 0.556 | 10 | 1.3E-02 |
| CA | -4.286 | 19.054 | 0.795 | 7 | 7.E-03 | -5.622 | 23.035 | 0.534 | 10 | 1.6E-02 |
| BT | -4.913 | 21.414 | 0.793 | 7 | 7.E-03 | -6.493 | 26.131 | 0.536 | 10 | 1.6E-02 |
| BZ | -7.177 | 29.632 | 0.789 | 7 | 8.E-03 | -9.495 | 36.307 | 0.502 | 10 | 2.1E-02 |
| IV | -8.742 | 35.561 | 0.795 | 7 | 7.E-03 | -10.200 | 39.055 | 0.522 | 10 | 1.8E-02 |
| VA | -7.717 | 31.762 | 0.796 | 7 | 7.E-03 | -11.402 | 43.221 | 0.513 | 10 | 1.9E-02 |
| OT | -11.640 | 46.425 | 0.778 | 7 | 9.E-03 | -15.393 | 56.906 | 0.485 | 10 | 2.5E-02 |
| MT | -12.748 | 50.085 | 0.782 | 7 | 8.E-03 | -16.251 | 59.626 | 0.476 | 10 | 2.7E-02 |
| PT | -13.387 | 52.267 | 0.785 | 7 | 8.E-03 | -16.590 | 60.668 | 0.471 | 10 | 2.8E-02 |
| HA | -15.436 | 59.896 | 0.790 | 7 | 8.E-03 | -20.104 | 72.926 | 0.490 | 10 | 2.4E-02 |
| DB | -20.611 | 78.986 | 0.783 | 7 | 8.E-03 | -25.071 | 89.656 | 0.438 | 10 | 3.4E-02 |

The data for each variable were first treated with linear regression analysis to predict the goodness-of-fit for our data. These datasets were evaluated further to assess their relative significance in terms of correlation strengths. The results of individual variables against M/W ratio, summarized in Table 6, indicate that the strongest relationship with M/W ratio is found from the run time data, followed by $R_s$ value and slope value. The changes in M/W ratio were fairly sensitive enough to alternate the trend of run time with a statistical significance. If the results are compared by the confidence interval of 95%, the M/W ratio is found to act most effectively on the determination of run time values. The tight relationship between the run time and M/W ratio is found to be significant from all investigated cases. Likewise, the M/W ratio is also found to be a critical variable to determine $R_s$ values; a total of 11 (WIM) and 7 (WTM) components are found to be affected significantly by M/W ratio ($p < 0.05$). In contrast, the effect of M/W ratio variation appears to be the least effective to describe their sensitivity. Their significant relationship is found in as many as six components of the WIM category, while only one case (AR) of WTM experiments appears to be significant with the M/W ratio. As a result, one may conclude that the use of the M/W ratio is a fairly effective tool, although one needs to be more cautious about its relationship between different variables that interact with the HPLC performance.

## CONCLUSION

In an effort to provide more pertinent options to CC analysis, we investigated all the optimal conditions of their separation based on ternary mobile phases. To this end, three types of ternary mobile phases (i.e., WIM, WTM, and WIT) were initially designed and tested in reference to a standard binary mixture (ACN:water). The results indicate that the separation of CCs can be archived in a narrow range of water composition (30 to 35%) in terms of maximum number of quantifiable CCs. The sensitivity of the method was evaluated by calibration slope values of all experiments. The results of this comparison showed that the sensitivity of lighter CCs is generally lower than or comparable to the reference binary (ACN:water) phase, while that of heavier CCs tends to improve significantly. The run time for the full separation of 15 CCs tends to increase, as water composition increases gradually. Nonetheless, this run time effect is helpful in achieving a better resolution for the peaks that were otherwise separated poorly. In a practical sense, the balance between run time and resolution factors ($R_s$) should be considered in a real situation. In addition, the relative patterns of $R_s$ were seen to differ slightly between the WIM and WTM phases. Besides, complete separation of peaks was attained rather scarcely among the heavy CCs, e.g., OT (11), MT (12), and PT (13), and some lighter CCs. Such problems were resolved in the course of experiments by increasing the water composition in the mobile phase to a maximum permissible degree. In conclusion, experiments conducted at the higher content of water (or lower M/W ratios) showed near optimal conditions for the separation of the aldehyde/ketone mixture at the cost of extended run time. In this study, the relative performance of ternary mobile phases for all different compositional settings was explained by their relationship with water content (or such variables as M/W ratio). As such, the results of this study can be used to provide a formula of mobile phase that can facilitate the analysis of CCs in both real field situations and under laboratory conditions.

## ACKNOWLEDGMENT

This study was supported by a National Research Foundation of Korea (NRF) grant funded by the Ministry of Education, Science and Technology (MEST) (No. 2009-0093848).

## References

1. de Carvalho, A.B., Kato, M., Rezende, M.M., de P. Pereira, P.A., and de Andrade, J.B. (2008) Determination of carbonyl compounds in the atmosphere of charcoal plants by HPLC and UV detection. *J. Sep. Sci*. **31,** 1686–1693.
2. Feng, Y., Wen, S., Chen, Y., Wang, X., Lü, H., Bi, X., Sheng, G., and Fu, J. (2005) Ambient levels of carbonyl compounds and their sources in Guangzhou, China. *Atmos. Environ*. **39,** 1789–1800.
3. Baez, A.P., Belmont, R., and Padilla, H. (1995) Measurements of formaldehyde and acetaldehyde in the atmosphere of Mexico City. *Environ. Pollut*. **89**, 163–167.
4. Weng, M., Zhu, L., Yang, K., and Chen, S. (2009) Levels and health risks of carbonyl compounds in selected public places in Hangzhou, China. *J. Hazard. Mater*. **164,** 700–706.
5. Vogel, M., Büldt, A., and Karst, U. (2000) Hydrazine reagents as derivatizing agents in environmental analysis – a critical review. *Fresenius* J. Anal. Chem. **366,** 781–791.
6. Bakeas, E.B., Argyris, D.I., and Siskos, P.A. (2003) Carbonyl compounds in the urban environment of Athens, Greece. *Chemosphere* **52,** 805–813.
7. Possanzini, M., Di Palo, V., Petricca, M., Fratarcangeli, R., and Brocco, D. (1996) Measurements of lower carbonyls in Rome ambient air. *Atmos. Environ.* **30,** 3757–3764.
8. Wang, B., Lee, S.C., and Ho, K.F. (2007) Characteristics of carbonyls: concentrations and source strengths for indoor and outdoor residential microenvironments in China. *Atmos. Environ.* **41,** 2851–2871.
9. Pang, X. and Mu, Y. (2006) Seasonal and diurnal variations of carbonyl compounds in Beijing ambient air. *Atmos. Environ.* **40** (2006) 6313–6320.
10. Ho, K.F., Lee, S.C., Louie, P.K.K., and Zou, S.C. (2002) Seasonal variation of carbonyl compound concentrations in urban area of Hong Kong. *Atmos. Environ.* **36,** 1259–1265.
11. Seaman, V.Y., Charles, M.J., and Cahill, T.M. (2007) A sensitive method for the quantification of acrolein and other volatile carbonyls in ambient air. *Anal. Chem*. **78,** 2405–2412.
12. Pal, R. and Kim, K.-H. (2007) Experimental choices for the determination of carbonyl compounds in air. *J. Sep. Sci.* **30,** 2708–2718.
13. Possanzini, M., Di Palo, V., Brancaleoni, E., Frattoni, M., and Ciccioli, P. (2000) A train of carbon and DNPH-coated cartridges for the determination of carbonyls from C1 to C12 in air and emission samples. *Atmos. Environ.* **34,** 5311–5318.
14. Kim, K.-H., Hong, Y.-J., Pal, R., Jeon, E.-C., Koo, Y.-S., and Sunwoo, Y. (2001) Investigation of carbonyl compounds in air from various industrial emission sources. *Chemosphere* **70,** 807–820.

15. Fung, Y.-S. and Long, Y. (2001) Determination of carbonyl compounds in air by electrochromatography. *Electrophoresis* **22,** 2270–2277.
16. Herrington, J.S., Fan, Z.-H., Lioy, P.J., and Zhang, H. (2007) Low acetaldehyde collection efficiencies for 24-hour sampling with 2,4-dinitrophenylhydrazine (DNPH)-coated solid sorbents. *Environ. Sci. Technol*. **41,** 580–585.
17. Possanzini, M., Tagliacozzo, G., and Cecinato, A. (2007) Simultaneous determination of formic acid and lower carbonyls in air samples by DNPH derivatization. *J. Sep. Sci*. **30,** 2460–2465.
18. Spada, N., Fujii, E., and Cahill, T.M. (2008) Diurnal cycles of acrolein and other small aldehydes in regions impacted by vehicle emissions. *Environ. Sci. Technol*. **42,** 7084–7090.
19. Liu, L.J.S., Dills, R.L., Paulsen, M., and Kalman, D.A. (2001) Evaluation of media and derivatization chemistry for six aldehydes in a passive sampler. *Environ. Sci. Technol*. **35,** 2301–2308.
20. Büldt, A. and Karst, U. (1999) N-methyl-4-hydrazino-7-nitrobenzofurazan as a new reagent for air monitoring of aldehydes and ketones. *Anal. Chem*. **71,** 1893–1898.
21. Possanzini, M., Di Palo, V., and Cecinato, A. (2004) Field evaluation of N-methyl-4-hydrazino-7-nitrobenzofurazan (MNBDH) coated silica gel cartridges for the measurement of lower carbonyls in air. *Chromatographia* **60,** 715–719.
22. Jakober, C.A., Charles, M.J., Kleeman, M.J., and Green, P.G. (2006) LC-MS analysis of carbonyl compounds and their occurrence in diesel emissions. *Anal. Chem.* **78,** 5086–5093.
23. Kim, K.-H. and Pal, R. (2010) Determination of acetaldehyde in ambient air: comparison of thermal desorption-GC/FID method with the standard DNPH-HPLC method. *Environ. Monit. Assess.* **161**, 295–299.
24. Uchiyama, S., Matsushima, E., Aoyagi, S., and Ando, M. (2004) Simultaneous determination of C1 C4 carboxylic acids and aldehydes using 2,4-dinitrophenylhydrazine-impregnated silica gel and high-performance liquid chromatography. *Anal. Chem.* **76,** 5849–5854.
25. Long, W.J. and Henderson, J.W. (2008) Rapid Separation and Identification of Carbonyls Compounds by HPLC. Aligent Technologies, Wilmington, DE. 5989-7483EN.
26. Ho, D.X. and Kim, K.-H. (2011) Effect of HPLC binary mobile phase composition on the analysis of carbonyls. *Environ. Monit. Assess*. DOI 10.1007/s10661-010-1779-7
27. Dolan, J.W. (2002) Peak Tailing and Resolution. LC•GC Europe. LC Resources, Walnut Creek, CA.
28. Supelco (1997) Data Sheet of Supelcosil LC-18 (P/N 58298) column.
29. Ochs, S. de M., Fasciotti, M., Barreto, R.P., de Figueiredo, N.G., Albuquerque, F.C., Massa, M.C.G.P., Gabardo, I., and Netto, A.D.P. (2010) Optimization and comparison of HPLC and RRLC conditions for the analysis of carbonyl-DNPH derivatives. *Talanta* **81,** 521–529.
30. Prieto-Blanco, M.C., Iglesias, M.P., López-Mahía, P., Lorenzo, S.M., and Rodríguez, D.P. (2010) Simultaneous determination of carbonyl compounds and polycyclic aromatic hydrocarbons in atmospheric particulate matter by liquid chromatography–diode array detection–fluorescence detection. *Talanta* **80,** 2083–2092.
31. Potter, W. and Karst, U. (1996) Identification of chemical interferences in aldehyde and ketone determination using dual-wavelength detection. *Anal. Chem.* **68,** 3354–3358.
32. Koivusalmi, E., Haatainen, E., and Root, A. (1999) Quantitative RP-HPLC determination of some aldehydes and hydroxyaldehydes as their 2,4-dinitrophenylhydrazone derivatives. *Anal. Chem.* **71,** 86–91.
